# Spontaneous pneumomediastinum in COVID‐19 patients: two cases

**DOI:** 10.1186/s43168-022-00144-8

**Published:** 2022-07-07

**Authors:** Ramazan Sivil, Fatih Selvi, Cihan Bedel, Mustafa Korkut

**Affiliations:** Department of Emergency Medicine, Antalya Training and Research Hospital, Health Science University, Antalya, 07100 Turkey

**Keywords:** Coronavirus disease 2019, Spontaneous pneumomediastinum, Computed tomography, Complication

## Abstract

Coronavirus disease 2019 (COVID-19) has become a massive epidemic affecting millions of people worldwide. The common radiological findings of COVID-19 are peripheral ground glass or consolidative opacities, but pneumomediastinum is a very rare finding of COVID-19, especially in patients not receiving mechanical ventilation support. Our aim was to present cases of spontaneous pneumomediastinum in two patients with COVID-19 and to discuss the potential mechanism underlying it.

## Background

Coronavirus disease 2019 (COVID-19) has become a massive epidemic affecting millions of people worldwide. Patients can apply with a clinical presentation ranging from asymptomatic infection to critical and fatal disease [1, 2]. Pneumomediastinum is associated with the existence of air in the mediastinum. In spontaneous pneumomediastinum (SPM), which is first described by Louis Hamman, patients frequently present to emergency departments with chest pain, shortness of breath, and subcutaneous emphysema [3]. The common radiological findings of COVID-19 are peripheral ground glass or consolidative opacities, but pneumomediastinum is a very rare finding of COVID-19, especially in patients not receiving mechanical ventilation support [4]. Our aim was to present cases of SPM in two patients with COVID-19 and to discuss the potential mechanism underlying it.

## Case presentation

### Case 1

**Fig. 1:**
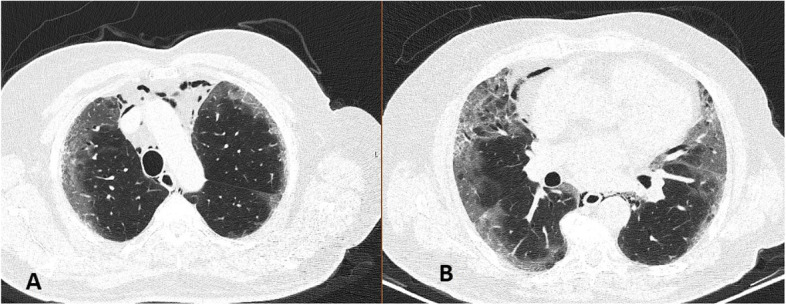
Chest CT obtained at presentation to the ED in case 1, demonstrating mediastinal air (**A**). It shows bilateral ground-glass opacities and parenchymal consolidation (**A**–**B**)

A 68-year-old male patient admitted to the emergency department with complaints of shortness of breath and chest pain. The patient had a history of hypertension and cerebrovascular disease. The patient was diagnosed with COVID-19 based on the SARS-CoV-2 polymerase chain reaction (PCR) test in another hospital where he presented with similar complaints about a week ago and was followed up at home with the prescription of oral favipiravir 2 × 600 mg after a loading dose of 2 × 1600 mg. Due to the gradual increase in shortness of breath at home, he presented to the emergency department again a week later. His general condition was moderate-poor. His body temperature was 36.7 °C, oxygen saturation was 75%, heart rate was 116 bpm, and blood pressure was 145/80 mmHg. The laboratory values of the patient were as follows: white blood cell count, 22.5 × 10^3^/mm^3^; C-reactive protein, 2.8 mg/L; and D-dimer, 2375 µg/L. The other values were insignificant. The patient’s saturation was 98% under 10 l/min oxygen support with a non-rebreather mask. The ordered unenhanced chest computed tomography (CT) of the patient showed diffuse air densities in the mediastinal region and an appearance consistent with pneumomediastinum. In addition, diffuse and patchy intense ground-glass density increases in the subpleural areas of both lungs, partial honeycomb lung appearance, and radiological appearance consistent with COVID-19 were present (Fig. 1a–b). The patient was followed up in the intensive care unit for SPM and COVID-19. The patient had a progression to acute respiratory distress syndrome (ARDS) with the current clinic. Despite aggressive medical therapy, inotropic support, and mechanical ventilation support, the patient died 1 week after hospitalization.

### Case 2

**Fig. 2:**
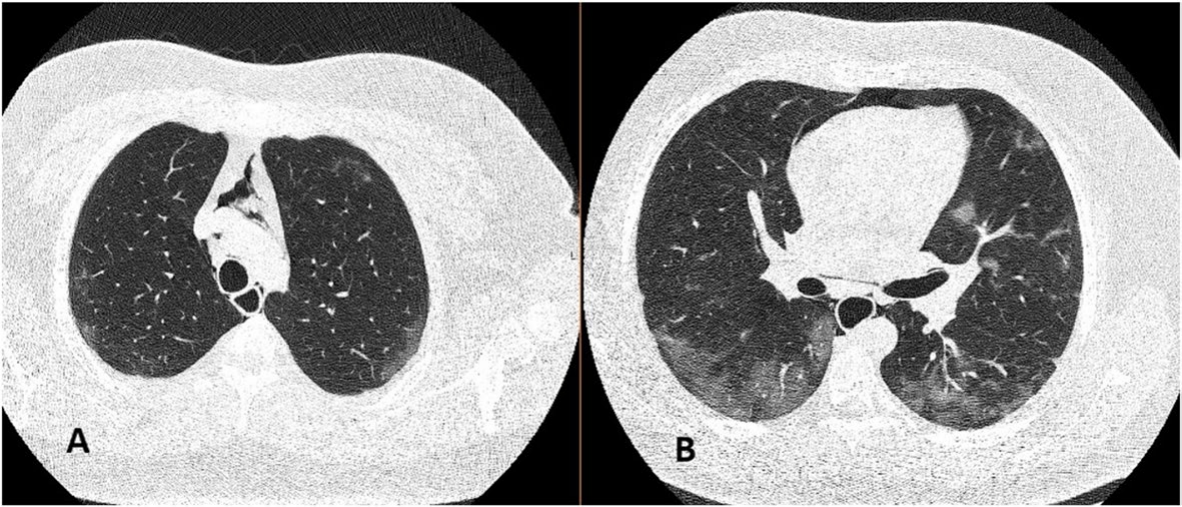
Chest CT obtained at presentation to the ED in case 2, demonstrating mediastinal air (**A**). It shows bilateral ground-glass opacities and parenchymal consolidation (**A**–**B**)

A 27-year-old male patient admitted with complaints of fever, cough, sore throat, and fatigue that started 5 days ago. On the second day of his complaints, he applied to another center and was diagnosed with COVID-19. On the third day of his treatment, he presented to our emergency department due to the increase in his sore throat and fatigue complaints. The patient’s oropharynx was normal, and the general condition was good. His body temperature was 37.5 °C, saturation was 94%, heart rate was 118/bpm, and blood pressure was 125/78 mmHg. The patient’s medical history was insignificant. He did not smoke or drink alcohol. The laboratory values of the patient were as follows: white blood cell count, 10.5 × 103/mm^3^; C-reactive protein, 57.6 mg/L; and D-dimer, 452 µg/L. The other values were insignificant. The unenhanced chest CT showed areas of diffuse patchy ground-glass infiltration in both lung parenchymal areas and an appearance suggestive of COVID-19 pneumonia. Free air densities were existence in the mediastinum, which were consistent with pneumomediastinum (Fig. 2a–b). The SARS-CoV-2 polymerase chain reaction (PCR) test of the patients was also positive. The patient was followed up with clinical findings and daily chest X-rays. He received oxygen support and oral favipiravir 2 × 600 mg following a loading dose of 2 × 1600 mg. The patient whose complaints regressed after 2 days of hospitalization was discharged.

## Discussion

SPM is a rare complication of COVID-19, with reported cases in patients with severe acute respiratory syndrome, influenza, and immune suppression [5]. Although the underlying mechanism for the development of SPM during respiratory tract infections has not been clearly explained, several mechanisms have been suggested to clarify this issue, one of which is known as the Macklin phenomenon. This phenomenon is characterized by alveolar rupture caused by alveolar damage due to direct viral infection or cytokine storm, with the consequent escape of air into the mediastinum through the peribronchial and perivascular sheaths [6].

Patients with SPM often present with symptoms such as shortness of breath, cough, chest, or cervical pain, and physical manifestations mainly include tachycardia, tachypnea, hypotension, and subcutaneous emphysema [7]. SPM is usually a self-limiting and benign condition. It may indicate a potential poor condition, as the prognosis depends on the treatment of the underlying condition [4–7]. Although SPM has been associated with poor clinical prognosis by deepening respiratory acidosis in patients with COVID-19, there is no consensus yet on the management of these patients [8]. The search for a treatment modality continues. However, currently, no treatment has been confirmed to be safe and effective in SPM patients with COVID-19, and the search for a COVID-19 treatment modality is ongoing to reduce transmission, improve outcomes, and reduce complications [9].

## Conclusion

Although SPM is a rare complication of COVID-19 pneumonia, emergency physicians should keep this clinical condition in mind.

## Data Availability

The datasets used or analyzed during the current study are available from the corresponding author on reasonable request.
